# The Mediating Role of Interoceptive Sensitivity in the Relationship between Physical Activity and Depression Symptoms in College Students

**DOI:** 10.3390/bs14070608

**Published:** 2024-07-18

**Authors:** Qian Sun, Xiaona Shen, Meiling Qi, Muhammad Suliman, Siyu Tian

**Affiliations:** 1 School of Physical Education, Shandong University, Jinan 266237, China; sunqian66@sdu.edu.cn; 2 School of Nursing and Rehabilitation, Shandong University, Jinan 250012, China; sxn11220305@163.com (X.S.); meiling.qi@sdu.edu.cn (M.Q.)

**Keywords:** interoceptive sensitivity, physical activity, depression, college students

## Abstract

A substantial body of evidence indicates that neurological, psychological, and behavioral health issues are profoundly linked to interoceptive sensitivity. The present study aimed to identify the effects of interoceptive sensitivity on the relationship between physical activity and symptoms of depression in Chinese college students. This study employed a cross-sectional design using convenience sampling. An online self-reported survey was distributed to college students in China. The participants’ interoceptive sensitivity, physical activity levels, and depressive symptoms were measured using the MAIA-2, IPAQ-SF, and PHQ-9, respectively. The mediating effect was tested via regression analysis and a parallel mediation model, with bootstrap confidence intervals for indirect effects. The results showed a significant negative correlation between physical activity and depression. A significant positive correlation was observed between physical activity and seven dimensions of interoceptive sensitivity. Conversely, interoceptive sensitivity exhibited a negative correlation with depression. The bootstrap mediation analysis showed that the “not distracting” and “trusting” dimensions of interoceptive sensitivity had significant indirect effects on the relationship between physical activity and depression, suggesting that physical activity might reduce depressive symptoms via these two interoceptive sensitivity dimensions. The findings suggest that interoceptive sensitivity should be integrated into therapeutic interventions, such as physical activity interventions, in the treatment of mental illnesses, particularly depression. Increasing physical activity levels, with a specific focus on enhancing interoceptive modulation, appears to be a promising approach for addressing depression in college students.

## 1. Introduction

Depression is the most debilitating mental illness and one of the top twenty-five global causes of burden [1]. A study conducted in the United States found that military-educated graduate students had six times higher depression rates than the general population [2]. The prevalence of depression in previous studies among college students was reported as highest in Brazil (62.93%), followed by the USA (33%), China (28.90%), Pakistan (10.67%), and the lowest (7.9%) in Australia [3,4]. Depressed students exhibit a broad range of psychosomatic symptoms, as well as social disruption, suicidal ideation, impaired cognition, and reduced quality of life [5]. Additionally, the existing studies highlight the potential impact of participating in physical activity on the severity of depression symptoms affecting college students [3]. Regarding the association between physical activity and depression, alternative therapies such as exercise interventions, compared to anti-depressant drugs, have well-established antidepressive benefits and no side-effects [6], reducing a multitude of depressive symptoms including perceived stress, anhedonia, low energy levels, rumination, despair, and low mood [7]. Furthermore, physical activity has been linked to improvements in cognition, quality of life, sleep, stress hormones, gut microbiota, and cardiorespiratory fitness among patients with depression [8]. In China, the exercise initiative campaigns and themes under the Sustainable Developmental Goal and National Fitness Plan play a crucial role in shaping people’s health behaviors and mental health [9,10]. A previous study highlights the moderating role of quality of life in fostering a healthier, happier, and more vibrant society through physical activity initiatives [11]. Given the potentially beneficial effects of adequate physical activity on alleviating depression symptoms within the context of attaining well-being through natural means [12], more attention should be paid to other factors mediating these two variables among college students.

Research indicates that different domains of neurological, mental, and behavioral disorders, including anxiety and depression, are associated with changes in the structure, function, or connectivity of the interoceptive neural network [13,14]. Interoception, referring to the awareness, perception, or sensitization of internal body sensations, comprises three independent dimensions: interoceptive sensitivity (primarily related to the self-perceived ability to recognize internal body signals, and assessing subjectively), accuracy (the precise detection of internal body sensations and assessing objectively), and awareness (the ability to identify, access, understand and respond appropriately to patterns of internal signals) [14]. Adequate interoceptive sensitivity involves the efficient understanding and interpretation of visceral afferent information through its eight dimensions: noticing, not distracting, not worrying, attention regulation, emotional awareness, self-regulation, body listening, and trusting, as assessed by the Multidimensional Assessment of Interoceptive Awareness, version 2 (MAIA-2) [15,16]. Conversely, hypo-interoceptive sensitivity can create internal states that are difficult to track, while hyper-interoceptive sensitivity may lead to overwhelming sensations [17]. Symptoms and correlates of depression, such as changes in appetite, weight fluctuations (including both starvation and obesity), sleep disorders, sexual dysfunction, fatigue, dizziness, headaches, and pain, are strongly linked to low interoception sensitivity, as evidenced by numerous studies [18,19]. Dysfunctional interoception is associated with changes in appetite, resulting in weight gain or loss, exhaustion, sluggishness, and the psychomotor slowing typically observed during depression [20]. The emerging paradigm linking interoception to affect, cognition, and mental health suggests that an imbalance in metabolism and energy regulation (allostasis), paired with a lack of interoceptive sensitivity, can lead to a multitude of depressive symptoms [21].

In recent years, new developments have highlighted the relationship between physical activity and interoceptive processes. Interoceptive sensitivity has been positively correlated with physical activity across various demographics, including children [22]. Previous research indicates that individuals with higher levels of interoceptive sensitivity perception have better control over their physical bodily load [23]. Similar results were observed in Chinese college students who underwent Tai Chi intervention, a notable mindfulness-based exercise that improved dimensions of interoceptive sensitivity and reduced anxiety levels [24]. A study on athletes and university students found that only the self-regulation and attention regulation components of the MAIA-2 mediated the relationship between mindfulness, physical activity, and mental skills [25]. Physical activity aids in regulating neural impulses within the interoceptive system [26]. Specifically, the top-down approach of consistently perceiving and analyzing bodily sensations to ensure allostasis is believed to emphasize the role of interoceptive sensitivity in regulating physical activity [27]. Additionally, enhancing physical activity can optimize the manipulation and integration of sensory input with precise affective responses through afferent signals of interoceptive sensitivity [26]. It is now well known that interoceptive dysfunctional processes play a role in the pathophysiology of mental and behavioral disorders, particularly depression [28]. Additionally, regulating peripheral physiology (including physical activity) through chemosensory techniques is a novel therapeutic approach to target symptoms of depression by modifying interoceptive signaling [29]. However, the existing research on the mediating role that interoceptive sensitivity plays in the relationship between physical activity and symptoms of depression remains limited and inconclusive.

In summary, studies have identified strong relationships between interoceptive sensitivity and symptoms of depression, as well as the beneficial effects of physical activity on improving interoceptive sensitivity. Thus, interoceptive sensitivity may serve as a potential mechanism to mediate the relationship between physical activity and symptoms of depression. However, no studies have yet examined the mediating role that interoceptive sensitivity plays in the context of physical activity and depression symptoms among college students. The present study aimed to investigate this mediating role. We postulated the following hypotheses: (a) there is a direct relationship between participation in physical activity and depression, and (b) interoceptive sensitivity dimensions (i.e., noticing, not distracting, not worrying, attention regulation, emotional awareness, self-regulation, body listening, and trusting) mediate the relationship between participation in physical activity and depression. Given that most research on this topic has not focused on college students, this study provides valuable insights into the interoceptive processes and their potential therapeutic implications in this population.

## 2. Materials and Methods

### 2.1. Study Design

We conducted a cross-sectional study using an online survey. Ethical approval was obtained from the Human Research Ethics Committee of the authors’ institution (2023-R-146). Completion and submission of the online survey implied consent to participate, as stated at the beginning of the survey.

### 2.2. Setting

This study was conducted among college students at a college in China. Recruitment took place between 5 November 2023 and 30 January 2024.

### 2.3. Participants and Sample Size

This study employed a convenience sampling strategy to invite college students to participate. Eligible participants were aged over 17 years and expressed interest in joining the study. Students with cognitive or significant vision impairments were excluded.

Potential participants were recruited using posters and an email sent by the College Deputy Vice-Chancellor (Administration), which informed students of current research projects and included a description of the purpose of the study, the inclusion and exclusion criteria, and a link to the online survey via a free online Chinese survey platform (https://www.wjx.cn, accessed on 10 October 2023).

We did not perform a prior calculation of the sample size and aimed to recruit as many participants as possible. In total, 800 college students were invited, and the questionnaire was self-administered by the participants. By the end of the recruitment period, 396 college students had participated, including 352 females (88.9%) and 44 males (11.1%). The average age of the participants was 19.33 years (SD = 1.02), with an age range from 17 to 23 years. The majority of participants (84%) lived in rural areas, while 16% resided in urban areas.

### 2.4. Measures

#### 2.4.1. Physical Activity Participation (Predictor)

The Chinese version of the International Physical Activity Questionnaire-Short Form (IPAQ-SF) was used to assess participants’ physical activity participation [30]. IPAQ scores can be processed and analyzed as either categorical or continuous data. Continuous scores measure energy expenditure in terms of metabolic equivalent of task (MET-minutes per week), while categorical scores classify participants into three physical activity levels: low, moderate, and high. The reliability of the IPAQ-SF was previously assessed among adults, showing an intraclass correlation coefficient of 0.67 in the current study.

#### 2.4.2. Depression Symptoms (Outcome Variable)

The Chinese version of the Patient Health Questionnaire-9 (PHQ-9) was used to assess participants’ depression symptoms over the previous two weeks [31]. This scale consists of nine items, each rated from 0 to 3. Scores of 4 or less indicate no depression, scores from 5 to 9 indicate mild depression, scores from 10 to 14 indicate moderate depression, scores from 15 to 19 indicate moderately severe depression, and scores from 20 to 27 indicate severe depression. In the current study, the PHQ-9 displayed a high Cronbach’s alpha (α = 0.94).

#### 2.4.3. Interoceptive Sensitivity (Mediator)

The Chinese version of the Multidimensional Assessment of Interoceptive Awareness- version 2 (MAIA-2) was used to assess participants’ interoceptive sensitivity [32]. The MAIA-2 contains 37 items rated on a 6-point Likert scale ranging from 0 (never) to 5 (always). The instrument includes eight subscales: (1) noticing, (2) not distracting (reverse scored), (3) not worrying (reverse scored), (4) attention regulation, (5) emotional awareness, (6) self-regulation, (7) body listening, and (8) trusting. A sum score can be calculated for each subscale. In the current study, the Cronbach’s alpha values for each subscale ranged from 0.75 to 0.91.

### 2.5. Statistical Analysis

The statistical software SPSS 24.0 was utilized for data analysis in this study. The Harman single-factor test was employed to determine the common method bias. Descriptive statistics were calculated and presented as frequencies (i.e., percentages) for categorical variables and as means and standard deviations for continuous variables. Pearson correlation analysis was used to explore the relationships between physical activity, different dimensions of interoceptive sensitivity (i.e., noticing, not distracting, not worrying, attention regulation, emotional awareness, self-regulation, body listening, and trusting), and depression symptoms.

Regression analysis was performed using the Hayes PROCESS macro (version 3.0) to test the study hypotheses, with physical activity serving as the independent variable (X), different dimensions of interoceptive sensitivity as the mediating variables (M), and depression as the dependent variable (Y). Before inclusion in the mediation analysis, all variables were standardized. The effect of 5000 bootstrap samples was assessed utilizing the PROCESS procedure, resulting in the determination of 95% bias-corrected confidence intervals (CIs). Effect sizes were deemed statistically significant when the confidence interval did not encompass zero. The effect achieved statistical significance at the predetermined level of *p* = 0.05.

## 3. Results

### 3.1. Common Method Bias Testing

The exploratory factor analysis revealed nine factors with eigenvalues exceeding one in the unrotated factor solutions. The total variance captured by the first factor with all measures included was 27.94%, which was below the established threshold of 40%. These results demonstrated that this study did not exhibit significant common method bias.

### 3.2. Descriptive Statistics and Correlation Analysis

Table 1 presents the correlation coefficients for physical activity, different dimensions of interoceptive sensitivity, and depression. The results indicate a significant negative relationship between physical activity and depression (*p* < 0.01), as well as positive correlations between physical activity and noticing, not distracting, attention regulation, emotional awareness, self-regulation, body listening, and trusting (*Ps* < 0.05). However, no significant relationship was observed between physical activity and not worrying. There were statistically significant negative correlations between depression and not distracting, not worrying, attention regulation, emotional awareness, self-regulation, body listening, and trusting (*Ps* < 0.01), but no significant correlation was found regarding noticing.

### 3.3. Regression Analysis of Physical Activity, Interoceptive Sensitivity, and Depression

Table 2 displays the regression coefficients of the model incorporating physical activity as the independent variable, depression as the dependent variable, and the different dimensions of interoceptive sensitivity as the mediating variables. The correlation analysis showed no significant correlation between noticing and depression or between not worrying and physical activity. Consequently, the interoceptive sensitivity dimensions included in the model as mediating variables were limited to not distracting, attention regulation, emotional awareness, self-regulation, body listening, and trusting.

**Figure 1:**
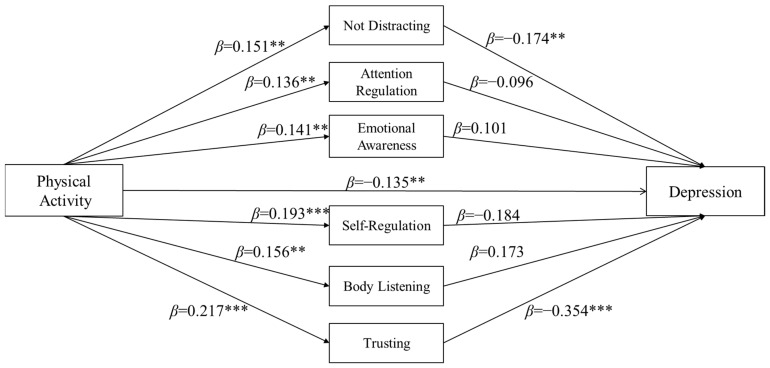
Mediation model of physical activity, interoception, and depression. ** *p* < 0.01; *** *p* < 0.001.

The findings indicated a statistically significant negative relationship between physical activity and depression, as evidenced by the negative regression coefficient (*β* = −0.245, *p <* 0.001). Furthermore, the negative effect of physical activity on depression persisted even after the inclusion of the mediating variables (*β* = −0.135, *p <* 0.01). Within the mediation model, physical activity demonstrated significantly positive effects on not distracting (*β* = 0.151, *p <* 0.01), attention regulation (*β* = 0.136, *p <* 0.01), emotional awareness (*β* = 0.141, *p <* 0.01), self-regulation (*β* = 0.193, *p <* 0.01), body listening (*β* = 0.156, *p <* 0.01), and trusting (*β* = 0.217, *p <* 0.001). However, the regression analysis revealed that attention regulation (*β* = −0.096, *p* > 0.05), emotional awareness (*β* = 0.101, *p* > 0.05), self-regulation (*β* = −0.184, *p* > 0.05), and body listening (*β* = 0.173, *p* > 0.05) did not have a significant impact on depression. Only the variables of not distracting (*β* = −0.174, *p <* 0.01) and trusting (*β* = −0.354, *p <* 0.001) were found to have significant negative effects on depression. The regression coefficients for each path in the model are displayed in Figure 1.

### 3.4. Mediation Analysis of Physical Activity, Interoceptive Sensitivity, and Depression

Table 3 illustrates the application of the bias-corrected 5000-sample bootstrap method in estimating the effect size of the model. The findings indicated that the model had a total effect of −0.245 (95% CI: −0.345, −0.145), with the total direct effect of physical activity on depression at −0.135 (95% CI: −0.231, −0.038) and the total indirect effect of interoceptive sensitivity at −0.110 (95% CI: −0.167, −0.060). The bootstrap analysis revealed that the 95% confidence intervals did not encompass 0, suggesting that the total effect, direct effect, and total indirect effect of the model were statistically significant.

Regarding the indirect effects specifically, the indirect effects of attention regulation (effect = −0.013; 95%CI: −0.036, 0.006), emotional awareness (effect = 0.014; 95%CI: −0.010, 0.044), self−regulation (effect = −0.036; 95%CI: −0.085, 0.003), and body listening (effect = 0.026; 95%CI: −0.002, 0.065) were found to be non-significant, with their 95% confidence intervals encompassing zero. Only the indirect effects of not distracting and trusting were statistically significant, as confirmed by the bootstrap analysis which indicated that their 95% confidence intervals did not contain zero. The effect size of not distracting was −0.026 (95%CI: −0.055, −0.007), while the effect size of trusting was −0.077 (95%CI: −0.127, −0.036). The research findings indicate that physical activity may alleviate symptoms of depression by influencing two interoceptive sensitivity dimensions: not distracting and trusting.

## 4. Discussion

### 4.1. The Relationship between Physical Activity, Interoceptive Sensitivity, and Depression in College Students

The findings indicated a negative correlation between physical activity and depression among college students, suggesting that individuals with higher levels of physical activity were less likely to exhibit symptoms of depression. These findings support hypothesis (a) and are consistent with the findings of a previous meta-analysis [6]. Other studies have also demonstrated that physical activity has a significant impact on mental health [11], serving as a valuable tool in promoting overall well-being and alleviating negative emotions in a natural manner [12,33].

Furthermore, this study revealed positive correlations between physical activity and seven interoceptive sensitivity dimensions, including noticing, not distracting, attention regulation, emotional awareness, self-regulation, body listening, and trusting. However, no significant correlation was found between physical activity and not worrying. These findings partially support the conclusions of Georgiou [22] and Wallman-Jones [26] regarding the potential of physical activity to improve interoceptive sensitivity.

Specifically, higher levels of physical activity facilitate heightened awareness of one’s health status, enabling individuals to discern between states of comfort and discomfort [34]. Moreover, given that the primary objective of physical activity among university students is to enhance or sustain their physical well-being [35], college students are inclined to attend to bodily sensations and not ignore any physical pain or discomfort experienced during exercise. Additionally, stress reduction and promotion of mental well-being and positive affect represent additional primary physical activity objectives for college students [32]. Consequently, engagement in physical activity enhances college students’ awareness of bodily sensations and the interplay between their physical and emotional states, thereby facilitating improved self-regulation. Furthermore, attaining heightened physical fitness through physical activity fosters a sense of bodily security and reliability among college students. Nevertheless, most students do not participate in the rigorous physical conditioning regimens typical of professional athletes, leading to a lack of significant worry about emotional distress deriving from physical pain or discomfort during routine physical exertion.

Regarding the relationship between interoceptive sensitivity and depression, the study’s findings indicate a significant negative correlation between depression and seven dimensions of interoceptive sensitivity, including not distracting, not worrying, attention regulation, emotional awareness, self-regulation, body listening, and trusting. In contrast, no correlation was found with the dimension of noticing. These results align with previous research conducted by Karanassios [36] and contribute to a deeper understanding of the relationship between interoceptive sensitivity dimensions and depressive symptoms. It is suggested that college students who actively pay attention to bodily discomfort, maintain focus on bodily sensations, cultivate an awareness of the connection between physical and emotional states, practice self-regulation, and trust their bodies are less prone to depression. Furthermore, as previous studies have highlighted, simply noticing bodily sensations without conscious regulation does not significantly alleviate depressive symptoms [37].

### 4.2. The Mediating Effect of Interoceptive Sensitivity between Physical Activity and Depression in College Students

The present study further identified that some dimensions of interoceptive sensitivity, namely, not distracting and trusting, may mediate the association between physical activity and depressive symptoms among college students. Therefore, hypothesis (b) was partially supported in this study. Previous research has demonstrated that physical activity can enhance prefrontal cortex activity, facilitating top-down mechanisms that aid in attention regulation and prevent distraction from extraneous stimuli [38]. Furthermore, de Jong [39] found that non-distraction could serve as a significant indicator of depressive symptoms, aligning with the current research outcomes and reinforcing the clinical relevance of non-distraction regarding depressive symptoms. Therefore, physical activity can enhance the physiological stimulation of college students, preventing them from being distracted by bodily pain and other negative feelings due to external stimuli. This kind of non-distraction from negative feelings may better relieve negative emotions than distraction, ultimately alleviating depressive symptoms in college students. Additionally, engaging in physical activity has been shown to positively impact the physical and mental well-being of college students, leading to improved self-perception and increased bodily trust [40,41]. Cultivating positive perceptions of one’s body has been identified as a beneficial intervention for alleviating depressive symptoms [42]. Therefore, fostering students’ confidence in their physical abilities through engagement in physical activities may contribute to the mitigation of depressive symptoms.

In summary, the findings indicate that college students who engage in physical activities can enhance their interoceptive sensitivity by reducing distraction and increasing bodily trust, which, in turn, improves the management of depressive symptoms. These findings reaffirm the notion that physical activity is essential in advancing the health-related Sustainable Development Goals and in furthering progress towards the attainment of the “Healthy China” objectives [9]. It is noteworthy that attention regulation, emotional awareness, self-regulation, and body listening, while closely linked to both physical activity and depressive symptoms, did not serve as mediating factors between the two. Although previous research has indicated that attention regulation, emotional awareness, self-regulation, and body listening can potentially alleviate depressive symptoms by addressing alexithymia and mood dysregulation [37], the findings of this study suggest that the impact of physical activity on these factors may not significantly contribute to reducing depressive symptoms in college students.

Specifically, based on the study’s findings, physical activity appears to facilitate the development of attention regulation, emotional awareness, self-regulation, and body listening. However, the impact of these factors on depressive symptoms was found to be non-significant. The non-significant effect may be because attention regulation, emotional awareness, self-regulation, and body listening, which are developed through physical activity, primarily contribute to the enhancement of positive emotions rather than to the alleviation of depressive symptoms. According to the Dual-Factor Model of Mental Health [43], positive and negative emotions may exist relatively independently, implying that an increase in positive emotions does not necessarily result in the elimination of negative emotions, such as depressive symptoms. Consequently, based on these findings, it can be inferred that improving attention regulation, emotional awareness, self-regulation, and body listening through physical activity may not yield the anticipated benefits in terms of alleviating depression.

### 4.3. Implications

The findings of this research hold significant implications. First, this study represents a pioneering investigation into the mediating role of interoceptive sensitivity in the relationship between physical activity and depression, offering a novel theoretical framework for understanding depressive symptoms. Second, the results highlight that physical activity influences depressive symptoms among college students via the not distracting and trusting dimensions of interoceptive sensitivity, suggesting potential avenues for targeted preventive interventions. Specifically, to prevent and alleviate depression, college students should be vigilant in acknowledging and addressing bodily pain or discomfort, promptly making the necessary adjustments, and cultivating a firm understanding of the safety and reliability of their bodies.

### 4.4. Limitations and Future Research Direction

The present study has certain limitations. First, its cross-sectional design precludes the establishment of causality between variables. Subsequent research endeavors may seek to elucidate the causal relationships between physical activity, interoceptive sensitivity, and depression via longitudinal tracking or experimental interventions. Second, this study used subjective scales to assess college students’ physical activity levels and psychological well-being. Future investigations could benefit from incorporating more objective measurement techniques, such as accelerometers, which are more reliable as physical activity indicators. Furthermore, given that the participants in this research were college students, it is necessary to investigate whether the results can be generalized to other demographic groups, such as adolescents and older individuals. Finally, confounders of depression, including the students’ age, year of study, or satisfaction with their major, may be significantly associated with depression [44], but this study did not identify the potential moderating role of these confounders.

## 5. Conclusions

This study introduces a novel theoretical framework for understanding depressive symptoms; it illustrates that, cultivated through physical activity, interoceptive sensitivity—specifically, not distracting and trusting—can serve as a resource to mitigate depressive mood among college students. This research investigates the correlation between physical activity, interoceptive sensitivity, and depression among college students, and has identified a significant negative relationship between physical activity and depression. Furthermore, this study substantiates that interoceptive sensitivity serves as a critical mediating factor in the relationship between physical activity and depression. Physical activity directly affects the depressive symptoms of college students; additionally, it does so via two dimensions of interoceptive sensitivity (not distracting and trusting), which play a mediating role in college students’ depressive symptoms.

## Figures and Tables

**Table 1:** Correlation matrix.

	1	2	3	4	5	6	7	8	9	10
1. Physical Activity	1									
2. Noticing	0.113 *	1								
3. Not Distracting	0.151 **	−0.313 **	1							
4. Not Worrying	0.019	−0.296 **	0.156 **	1						
5. Attention Regulation	0.136 **	0.452 **	−0.388 **	−0.135 **	1					
6. Emotional Awareness	0.141 **	0.458 **	−0.272 **	−0.123 *	0.768 **	1				
7. Self-Regulation	0.193 **	0.451 **	−0.167 **	−0.017	0.675 **	0.793 **	1			
8. Body Listening	0.156 **	0.416 **	−0.220 **	−0.083	0.718 **	0.721 **	0.818 **	1		
9. Trusting	0.217 **	0.355 **	−0.030	0.037	0.547 **	0.605 **	0.765 **	0.722 **	1	
10. Depression	−0.244 **	−0.035	−0.180 **	−0.181 **	−0.162 **	−0.178 **	−0.293 **	−0.211 **	−0.383 **	1
*M*	1071.496	2.493	3.189	2.583	2.075	2.242	2.297	2.164	2.404	4.493
*SD*	1027.466	1.001	0.906	0.499	0.893	0.97	0.96	0.903	0.987	4.734

* *p* < 0.05; ** *p* < 0.01.

**Table 2:** Regression analysis of physical activity, interoceptive sensitivity, and depression.

Outcome Variables	Predictive Variables	Goodness-of-Fit Indices			Regression Coefficient and Significance	
		*R*	*R* ^2^	*F*	*β*	*t*
Depression	Physical Activity	0.244	0.059	23.155	−0.245	−4.812 ***
Not Distracting	Physical Activity	0.151	0.023	8.282 **	0.151	2.929 **
Attention Regulation		0.136	0.019	6.944 **	0.136	2.635 **
Emotional Awareness		0.141	0.020	7.417 **	0.141	2.723 **
Self-Regulation		0.193	0.037	14.235 ***	0.193	3.773 ***
Body Listening		0.156	0.024	9.176 **	0.156	3.029 **
Trusting		0.217	0.047	18.127 ***	0.217	4.268 ***
Depression	Physical Activity	0.462	0.213	13.980 ***	−0.135	−2.743 **
	Not Distracting				−0.174	−3.244 **
	Attention Regulation				−0.096	−1.157
	Emotional Awareness				0.101	1.123
	Self-Regulation				−0.184	−1.763
	Body Listening				0.173	1.881
	Trusting				−0.354	−4.572 ***

** *p* < 0.01; *** *p* < 0.001.

**Table 3:** The mediation analysis of physical activity, interoceptive sensitivity, and depression.

Effect	Standardized Coefficient	Bootstrap *SE*	Bootstrap 95%CI Lower Limit	Bootstrap 95%CI Upper Limit
**Total effect**	−0.245	0.051	−0.345	−0.145
**Direct effect**	−0.135	0.049	−0.231	−0.038
**Total indirect effect**	−0.110	0.027	−0.167	−0.060
Not distracting	−0.026	0.013	−0.055	−0.007
Attention regulation	−0.013	0.010	−0.036	0.006
Emotional awareness	0.014	0.014	−0.010	0.044
Self-regulation	−0.036	0.023	−0.085	0.003
Body listening	0.026	0.016	−0.002	0.065
Trusting	−0.077	0.023	−0.127	−0.036

## Data Availability

The data presented in this study are available from the corresponding author upon request.
